# Impact of a Serious Game (#RedPingüiNO) to Reduce Facial Self-Touches and Prevent Exposure to Pathogens Transmitted via Hands: Quasi-Experimental Intervention

**DOI:** 10.2196/45600

**Published:** 2023-06-30

**Authors:** Marta Arévalo-Baeza, Alejandro Viuda-Serrano, Carmen Juan-Llamas, Pablo Sotoca-Orgaz, Iván Asín-Izquierdo

**Affiliations:** 1 Department of Education Sciences Faculty of Medicine and Health Science Universidad de Alcalá Alcalá de Henares, Madrid Spain; 2 Department of Biodiversity, Ecology and Evolution (Biomathematics) Faculty of Biological Sciences Universidad Complutense de Madrid Madrid Spain; 3 Physical Performance and Sports Research Center Department of Sports and Computer Sciences, Faculty of Sport Sciences Pablo de Olavide University Seville Spain; 4 Department of Biomedical Sciences Faculty of Medicine and Health Sciences Universidad de Alcalá Alcalá de Henares, Madrid Spain

**Keywords:** self-touching, face, serious game, health, pathogen transmission, hazard scenarios, body language

## Abstract

**Background:**

After the COVID-19 pandemic, society has become more aware of the importance of some basic hygienic habits to avoid exposure to pathogens transmitted via hands. Given that a high frequency of touching mucous membranes can lead to a high risk of infection, it is essential to establish strategies to reduce this behavior as a preventive measure against contagion. This risk can be extrapolated to a multitude of health scenarios and transmission of many infectious diseases. #RedPingüiNO was designed as an intervention to prevent the transmission of SARS-CoV-2 and other pathogens through the reduction of facial self-touches by thoughtfully engaging participants in a serious game.

**Objective:**

Facial self-touches should be understood as behaviors of limited control and awareness, used to regulate situations of cognitive and emotional demands, or as part of nonverbal communication. The objective of this study was to ensure that participants become aware of and reduce these behaviors through a game of self-perception.

**Methods:**

The quasi-experimental intervention was applied to 103 healthy university students selected by convenience sampling and put into practice for 2 weeks, with 1 control group (n=24, 23.3%) and 2 experimental groups (experimental group with no additional social reinforcement interventions: n=36, 35%; experimental group with additional social reinforcement interventions: n=43, 41.7%). The objective was to improve knowledge and perception and reduce facial self-touches to prevent exposure to pathogens transmitted via hands not only in health multihazard scenarios but also in ordinary circumstances. The ad hoc instrument used to analyze the experience consisted of 43 items and was valid and reliable for the purpose of this study. The items were divided into 5 blocks extracted from the theoretical framework: sociological issues (1-5); hygiene habits (6-13); risk awareness (14-19); strategies for not touching the face (20-26); and questions after the intervention (27-42), designed as a postintervention tool assessing the game experience. Validation of the content was achieved through assessment by 12 expert referees. External validation was performed using a test-retest procedure, and reliability was verified using the Spearman correlation.

**Results:**

The results of the ad hoc questionnaire, which were analyzed using the Wilcoxon signed-rank test and McNemar index to identify significant differences between test and retest for a 95% CI, showed that facial self-touches were reduced (item 20, *P*<.001; item 26, *P*=.04), and awareness of this spontaneous behavior and its triggers increased (item 15; *P*=.007). The results were reinforced by qualitative findings from the daily logs.

**Conclusions:**

The intervention exhibited a greater effect from sharing the game, with interactions between people; however, in both cases, it was helpful in reducing facial self-touches. In summary, this game is suitable for reducing facial self-touches, and owing to its free availability and design, it can be adapted to various contexts.

## Introduction

### Background

The global pandemic caused by COVID-19 forced the World Health Organization and health care authorities to establish a series of fundamental measures to prevent the spread of infection, which have modified social behaviors [1,2]: social distancing, use of masks, frequent handwashing, and avoiding touching the face. Although the available epidemiological data and studies on environmental transmission factors have not defined fomite-mediated transmission through hand contact with the mucous membranes of the facial T-zone (eyes, nose, and mouth) as the main route, the risk of infection via this route by SARS-CoV-2 and other pathogens remains, as revealed by Marquès and Domingo [3], and rapid and direct recontamination can occur despite handwashing [4,5]. The survival of SARS-CoV-2 and other pathogens on surfaces is not known with accuracy, but we can take the influenza virus as a point of reference, which can survive for up to 10 minutes on our hands [6]. We can touch the mucous membranes of our face every 2 minutes [7], with a self-touching frequency of up to 50.06 times per hour [8], especially on the mouth, followed by the nose and eyes [9]. The frequency of hand-to-face contact shows great diversity; appears to be context dependent; is influenced by cognitive, attention, and emotional demands; and exhibits substantial individual and locational differences. It is difficult to establish an average [5,10], but given that a high frequency of touching the mucous membranes can lead to a high risk of infection, it seems essential to establish strategies to reduce this behavior as a preventive measure against contagions [11-13]. More importantly, this risk can be extrapolated to a multitude of health scenarios and the transmission of other infectious diseases.

Spontaneous facial self-touches (sFST) are defined as touches on one’s own body, from the hands to the face, which include movements such as rubbing, scratching, stroking, or leaning on the hands, and which involve a proprioceptive activation of joints, muscles, and connective tissue, being able to activate skin sensitivity in a more or less conscious way, as pointed out by Spille et al [12]. These authors emphasize the importance of different triggers of sFST, distinguishing self-touches by self-regulation in situations where complex cognitive processes occur; by regulation of the intensity of emotions (anxiety, tension, clumsiness, uncertainty, discomfort, pleasure, and surprise); and finally, by the manifestation of emotions not expressed through words, which can be understood from nonverbal communication. Thus, we grouped the triggers of sFST by distinguishing compensation touches (as a manifestation of underlying emotions to adapt to a given context); warning touches (with prior warning such as itching, perspiration, tingling, hair brushing, imperfections, or lacrimation); and communicative gestures (without prior warning, with a triggering situation, and learned gestures, such as a hand on the chin while listening or waiting).

To reduce sFST, we can apply heuristics through specific interventions by avoiding errors of omission with signals and reminders; emphasizing the use of “if-then” plans; applying substitute or alternative behaviors; and developing an adequate and progressive reorganization of the brain [14], a process that requires observation and becoming aware of the trigger from a conditional strategy [15]. It could also be treated by consciously learning each touch action; differentiating it from its usual response chain; and replacing it with another action [16], such as using a handkerchief or touching another area of the face that is not a mucous membrane. In summary, the leading techniques for changing habits and behaviors seem to be repetition, the use of instructions and signals, action planning, the provision of instructions regarding how to carry out the behavior, and the establishment of behavioral objectives in the search for these self-control behaviors.

### Objectives

This study was based on the serious game. This concept is understood as a carefully planned playful framework, the main purpose of which is to provide not only the entertainment itself but also an engaging context that involves people who are playing in the learning process [17-19]. Research has resorted to the use of serious games to address health care content, in the context of training, preventing disease, rehabilitating, or seeking to improve quality of life and well-being [20-22]. A serious game like the one analyzed in this study is a tool with complex development but simple intervention, which favors its use in various contexts, such as health institutions, hospitals, community centers, schools, or similar entities, and by the entire population, adapting the research procedures and design to the characteristics of the participants. This intervention does not require complex procedures, tools, techniques, or specialized software; therefore, it is accessible, efficient, and possibly helpful in reducing and perceiving the behavior of not touching the face. The #RedPingüiNO methodological proposal is an intervention based on a serious game with the main objective of reducing facial self-touches as a preventive measure against exposure to pathogens transmitted via hands, such as SARS-CoV-2 and others. The foundation of its design is underpinned by the multidisciplinary theoretical framework, focused on a playful, engaging, and viral proposal that uses metaphors, humor, and a scoring system as drivers of the proposed game, using digital resources designed ad hoc that can be adapted to different contexts. Therefore, the objective of this study was to analyze the effects of the #RedPingüiNO intervention on the awareness and reduction of facial self-touches, especially in the T-zone, based on self-perception, given that a serious game seeks to positively impact the habits of participants.

## Methods

### Research Design

**Figure 1:**
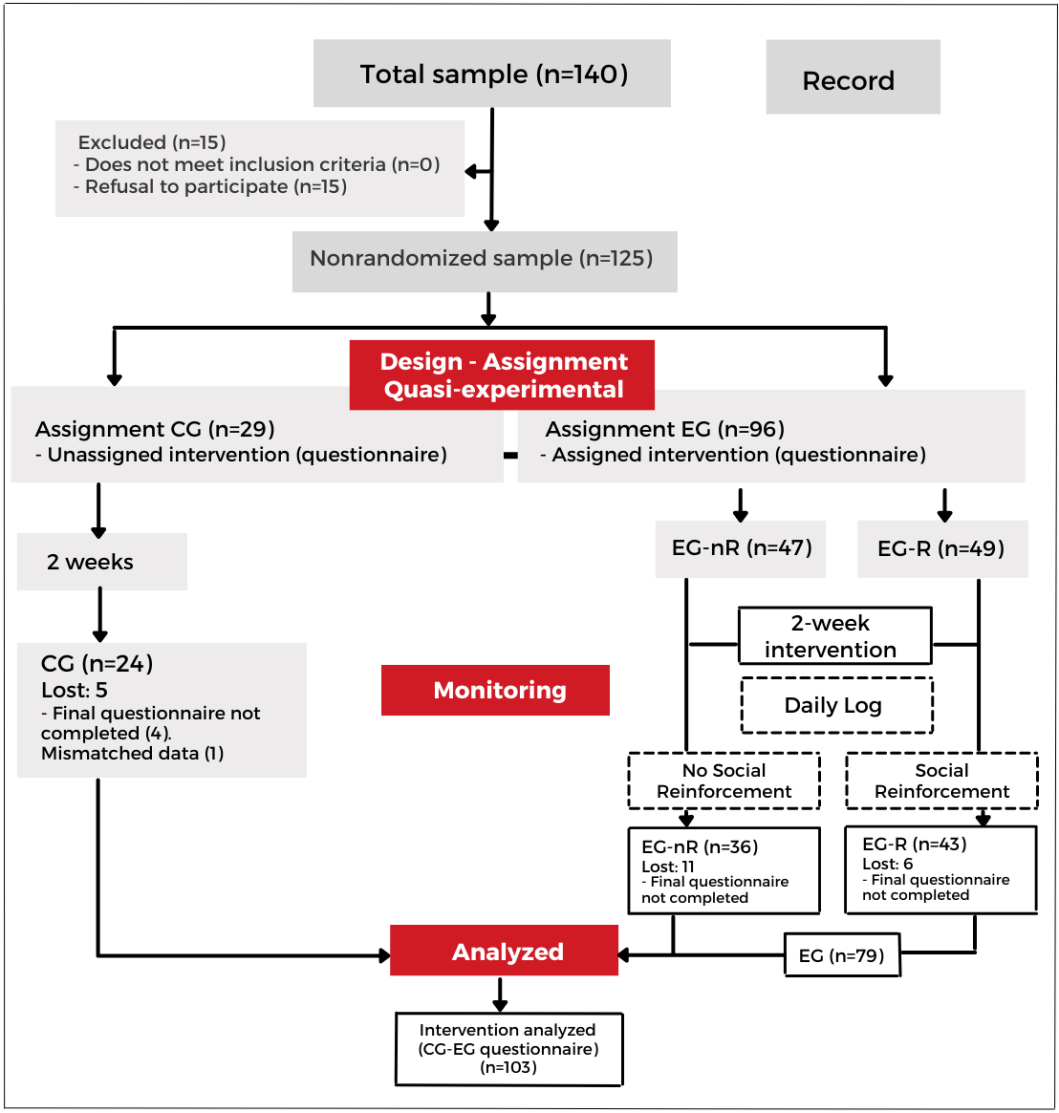
Flowchart of the intervention. CG: control group; EG: experimental group; EG-nR: experimental group with no additional social reinforcement interventions; EG-R: experimental group with additional social reinforcement interventions.

To design the research and the serious game, we have used Game-Based Intervention Reporting Guidelines given by Warsinsky et al [23]. Guidelines on conceptual issues, contributions of the study, related concepts, and definitions were taken into account; serious games were defined (*Introduction* section), the research stream was described (*Methods* section), the contribution of this study was clarified (*Discussion* section), and only core concepts based on the extant literature were used throughout the text. This study used a quasi-experimental design of involving a nonequivalent control group (CG) and a mixed approach. It is not possible to guarantee random assignment of the participants to the groups, but the educational center provides a predetermined distribution, ensuring limited homogeneity. This methodology is suitable for studying the potential causal effects of an intervention in open situations. The flowchart of the intervention is presented in Figure 1.

### Serious Game Design

#RedPingüiNO is a project based on a serious game that aims to reduce the spread of COVID-19 and other diseases through strategies to avoid the act of touching the face with one’s hands. The game uses penguins as protagonists because of their anatomy (they cannot touch their faces with their hands) and their social nature (collective and community behavior). The intervention platform has materials available in 8 languages. The game consists of associating sound onomatopoeias with behaviors: “No!” if the T-zone of the face is touched; “Ouch!” if another part of the face is touched; and “Bravo!” if the face is not touched at all. Descriptors and rankings are presented based on the daily frequency of self-touches (Figure 2). The rules of #RedPingüiNO were published on the #RedPingüiNO website [24].

**Figure 2:**
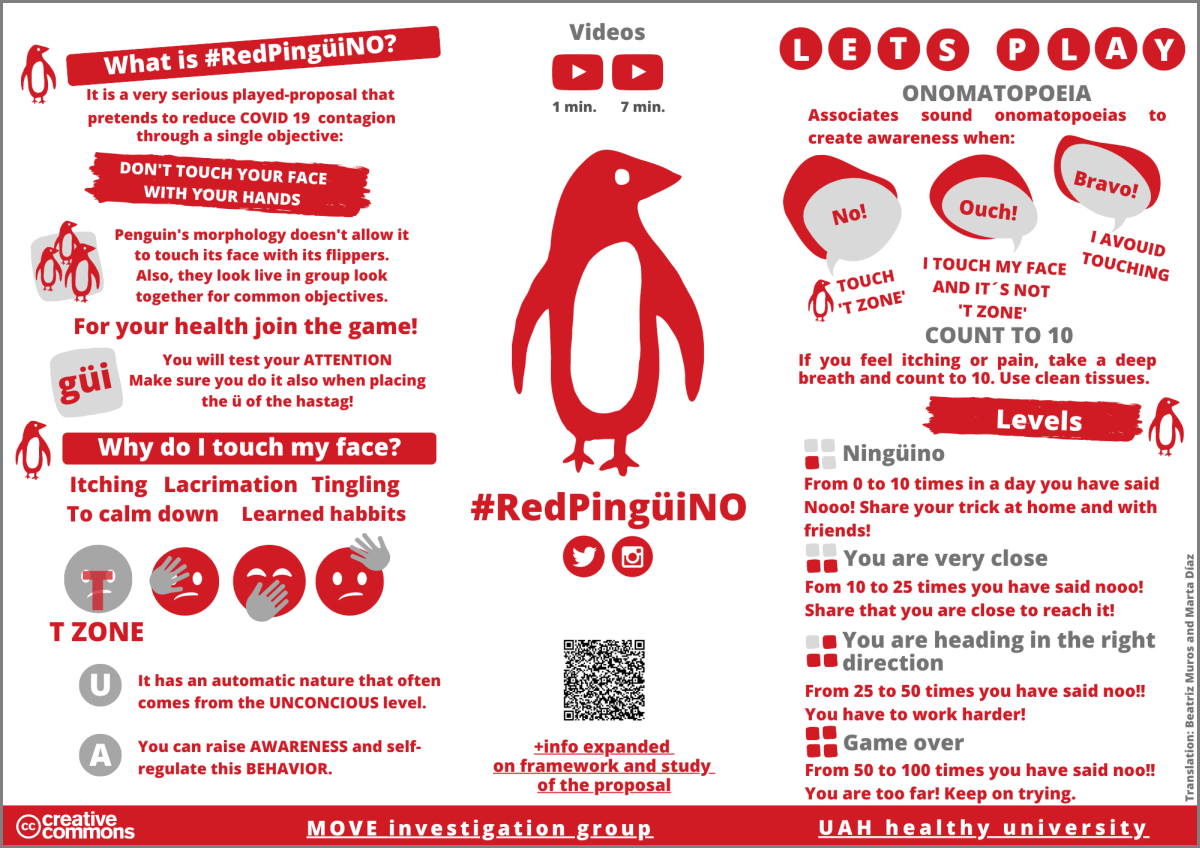
Infographics and #RedPingüiNO website.

### Participants

A total of 125 students taking the Primary Education Teaching Degree course from a public university in Madrid participated in this research. The sample was selected by convenience sampling with 103 healthy participants who completed the study, comprising 74 (71.8%) women and 29 (28.2%) men; 22 (21.4%) dropped out during the intervention, as they did not complete 80% of the proposed activities. The average age was 22.76 (SD 2.78) years and gender differences in the sample were due to the specific characteristics of the chosen degree, with a larger female representation. The participants were distributed into three groups via the use of the Telegram app (Telegram Messenger Inc): (1) the CG (n=24); (2) the experimental group with no additional social reinforcement (EG-nR; n=36); and (3) the experimental group with additional social reinforcement (EG-R; n=43). Participation in the study was voluntary and anonymous and required informed consent.

### Ethics Approval, Informed Consent, and Participation

This study was approved by the Ethics Committee of the Universidad de Alcalá (reference: CEI/HU/2020/46). The research complied with all national regulations and followed the tenets of the Declaration of Helsinki. The data of the participants were treated confidentially, and no personal information was accessed. Privacy was respected, and no personal information has been published. All the participants provided informed consent.

### Instruments

An ad hoc self-administered questionnaire about facial self-touches and the game #RedPingüiNO was administered to the participants using an easy survey platform before and after the intervention (the items in the instrument are listed in Multimedia Appendix 1). The instrument was made up of 43 items that were divided into 5 blocks and prepared from the following theoretical framework: (1) sociological issues (1-5), (2) hygiene habits (6-13), (3) risk awareness (14-19), (4) strategies for not touching the face (20-26), and (5) questions after the intervention (27-42). The fifth block was designed as a postintervention questionnaire and was administered only to the participants of the experimental groups (EGs). The items were closed ended, except for 1 open-ended question that collected considerations regarding the game (item 43).

The content validation of the questionnaire was conducted by expert judges [25]. A total of 12 judges (8 women and 4 men) assessed the content and form of the questionnaire. Their professional experience ranged from 11 to 35 years in scientific research related to the study, coming from various universities and heterogeneous fields of interest (body language, health, psychology, and didactics). The experts performed an overall assessment of the questionnaire and rated the degree of adequacy (relevance and wording). They were also asked about a set of aspects associated with the elaboration processes (appearance, clarity and length of the instructions, item format, and question order); data collection (completion time); and data interpretation (objectivity and absence of biases). The psychometric analysis of the content validity criteria of the instrument was performed using the V coefficient given by Aiken [26], considering the proportion of judges who express a positive assessment as a criterion for revising or eliminating items. An intervention pilot test and the registration were conducted by 4 teachers over a period of 5 days, during which they played the game and provided their assessments.

The participants of the EGs had to complete a daily self-compliance record of occurrence with 6 questions about the evolution of the intervention using the Microsoft Forms (Microsoft) tool and had to provide the following via Telegram: anonymous identification; frequency, prevention, and main reason for self-touches; motivation; and additional information. In the web space created for the EG-R, social reinforcement actions were carried out, freely sharing, among the participants, experiences during the intervention, daily achievements, difficulties, and additional information.

### Procedures

This research was coordinated and developed by faculty members and research staff who specialized in body language, with at least 10 years of experience. The questionnaires were administered by evaluators who were unaware of which group the participants belonged to, and the participants were unaware of the existence of other intervention groups.

The participants continued their daily routines throughout the intervention without supplementation of any other activity. The intervention was carried out for 2 weeks (November 23, 2020, to December 7, 2020) during the school term to foster the existence of a normalized context and to avoid the effect of holiday periods on the variation of routines. During this period, an attempt was made to modify the behavior of facial self-touches of the participants of the 2 EGs, EG-nR and EG-R, compared with that of the participants of the CG through the use of serious play. The reminders, sent via Telegram, occurred at the beginning of the day and around lunch time. At the end of the afternoon, a link to the registry was sent. The social reinforcement in the EG-R was in the form of praise, support, and approval, with tangible rewards and individual and collective reinforcement. The communication strategies used included cooperation, participation, interaction, and comparison.

### Data Analysis

Statistical analyses were performed using SPSS package (version 25.0; IBM Corp). The types of variables in the questionnaire, namely, categorical and dichotomous, made it necessary to use nonparametric tests. To study the reliability of the questionnaire, different statistics were used to assess test-retest reliability. To analyze whether there were statistically significant differences between the answers given to the same items in the test and retest, the Wilcoxon signed-rank test was used, except for dichotomous variables, which were analyzed using the McNemar index. In addition, the degree of correlation between the test and retest items was determined using the Spearman rank correlation coefficient for nonparametric samples. To demonstrate the reliability of items 27 to 42, which were answered only by the participants of the EGs, the Cronbach **α** was used. Furthermore, to analyze the significant differences between the different groups, the following was used: for groups taken 2 by 2, the Mann-Whitney *U* test for 2 independent samples and (2) to compare the 3 groups, the Kruskal-Wallis test for 3 independent samples. Finally, content analysis was used to analyze the open-ended questions in the questionnaire [27].

## Results

### Reliability and Validity of the Instrument

The V coefficient by Aiken [26], which was used to determine validity, was interpreted using the score method [28], and it showed a very positive and adequate result for the use of the questionnaire. This procedure was developed based on the expert method, assuming a 95% CI (*P*=.05). The value of the test was V=0.99 for the adequacy of the items and V=0.96 for the wording, with minimum values of 0.88 and 0.87, respectively. Table 1 shows the reliability of the instrument for items 6 to 26. The test-retest values were adequate because they were .05; therefore, it was not necessary to remove any of the items. The Spearman rank test for nonparametric samples confirmed these results, because no correlations .05 were observed.

Items 12 and 13 of the yes or no responses regarding the frequency of facial self-touches with a mask and knowledge of the facial T-zone did not show statistically significant differences (*P*=.45 and *P*=.53, respectively). The participants of the CG showed a higher frequency of facial self-touches with a mask (13/24, 54%), and 75% (18/24) knew about the facial T-zone before filling out the questionnaire. The reliability of the instrument for items 27 to 42 related to the intervention was analyzed using the Cronbach α (.723). The Cronbach α coefficient showed a value .70, which is the usual acceptance limit [29]. In summary, the questionnaire was valid, reliable, and therefore suitable for use.

**Table 1:** Reliability analysis of the questionnaire for the control group (n=24).

Construct and item		Test values, mean (SD)	Retest values, mean (SD)	Wilcoxon sign-rank test or McNemar index (*P* values)	Spearman rank coefficient (*P* values)
**Hygiene habits**					
	6	2.63 (0.92)	2.71 (0.81)	.21	.80
	7	2.33 (0.70)	2.54 (0.78)	.36	.46
	8	2.92 (1.06)	2.79 (1.06)	.16	.54
	9	2.67 (0.82)	2.58 (0.83)	.77	.46
	10	2.83 (0.87)	2.88 (0.74)	.76	.70
	11	3.92 (0.41)	3.83 (0.38)	.10	.35
**Risk awareness**					
	14	3.54 (0.51)	3.42 (0.58)	.08	.81
	15	3.63 (0.49)	3.54 (0.51)	.37	.41
	16	3.58 (0.58)	3.58 (0.50)	>.99	.23
	17	3.42 (0.58)	3.54 (0.51)	.16	.54
	18	3.58 (0.50)	3.58 (0.50)	.76	.33
	19	2.17 (0.64)	2.38 (0.65)	.16	.73
**Strategies for not touching the face**					
	20	1.83 (0.70)	2.04 (0.75)	.21	.65
	21	3.54 (0.51)	3.54 (0.51)	.48	.46
	22	3.54 (0.51)	3.38 (0.49)	.56	.26
	23	3.71 (0.55)	3.46 (0.51)	.25	.38
	24	2.96 (0.69)	2.75 (0.74)	.48	.47
	25	3.42 (0.65)	3.29 (0.55)	.16	.60
	26	2.46 (0.66)	2.50 (0.78)	.54	.33

### Hygiene Habits, Risk Awareness, and Strategies to Avoid Touching the Face

The significant pre- and postintervention differences in the participants of the EGs in terms of hygiene habits, risk awareness, and strategies to avoid touching the face are shown in Table 2. As Table 1 shows that there are no statistically significant differences in the CG, this information is omitted from Table 2.

Hygiene habits showed significant differences (*P*<.05) at pre- and postintervention time points in several items, as shown in Table 2. The 2 EGs showed coincident positive significant differences in item 9, which is related to the disinfection of objects. Item 6, regarding the frequency of handwashing, establishes a significant positive difference in the EG-R and a negative difference in the EG-nR. In addition, the participants of the EG-nR improved the behavior of avoiding touching the facial T-zone (item 7). Items 12 (EG-R, *P*>.99; EG-nR, *P*=.73) and 13 (EG-nR; *P*>.99) did not show significant differences at pre- and postintervention time points, with the exception of the EG-R for item 13, knowledge of the facial T-zone (*P*=.01). The participants of the EGs showed a higher frequency of facial self-touches with a mask (45/79, 57%), and 77% (61/79) knew about the facial T-zone before filling out the questionnaire. Risk awareness establishes pre- and postintervention differences (EG-nR and EG-R) in item 19, showing a perception toward greater ease in avoiding the behavior of touching the face. Moreover, the EG-nR showed higher values in the perception of the risk of touching the face (item 15).

By contrast, regarding the strategies for not touching the face, as the main objective of this study, consistent significant differences were observed. In the pre- and postintervention analysis, both EGs showed a reduction in the perception and frequency of facial self-touches (items 20 and 26). However, the EG-R established a positive significant difference in the perception of the magnitude of this behavior (item 26); this result is possibly related to an increase in perceptive capacity. In addition, a clear improvement was observed in the perception of the possibility of reducing facial self-touches with training and the importance of awareness of the behavior among the participants of EG-nR (items 22 and 23) and in seeking a simple solution to avoid a risk behavior among the participants of EG-R (item 24). The significant differences between the groups are shown in Table 3.

Regarding hygiene habits, there were differences between the CG and the 2 EGs, and the comparison of the 3 groups in relation to the ability of avoiding touching the facial T-zone favored the EGs (item 7).

With respect to risk awareness, positive significant differences were only observed between the CG and EG-nR, the CG and EG, and among the 3 groups in 2 items, 15 and 16, related to touching the face and handwashing. Finally, regarding the strategies for not touching the face, differences were observed between the CG and the 2 EGs, with higher rates observed in the EGs. These differences were observed for items 20, 24, and 25 in the EG-nR; for all items except 21 in the EG-nR; and for all except 21 and 23 in the full EG. In addition, differences were observed among the 3 groups for items 20 and 24. No significant differences were observed between the 2 EGs in any section.

**Table 2:** Significant differences between pre- and postintervention measures in the experimental groups.

Construct and item		Experimental group with no social reinforcement (n=36)			Experimental group with social reinforcement (n=43)		
		Test values, mean (SD)	Retest values, mean (SD)	Wilcoxon sign-rank test (*P* values)	Test values, mean (SD)	Retest values, mean (SD)	Wilcoxon sign-rank test (*P* values)
**Hygiene habits**							
	6	2.67 (1.06)	2.58 (1.02)	.005^a^	2.49 (0.88)	2.79 (0.77)	.04^a^
	7	2.56 (0.65)	2.94 (0.79)	.008^a^	2.65 (0.84)	3.02 (0.67)	.08
	8	3.03 (0.97)	3.03 (0.81)	.93	2.72 (1.12)	2.74 (0.93)	.85
	9	2.44 (0.81)	2.92 (0.73)	.001^a^	2.58 (0.98)	2.98 (0.96)	.03^a^
	10	2.72 (0.85)	2.94 (0.63)	.28	2.93 (0.83)	3.16 (0.92)	.10
	11	3.75 (0.50)	3.58 (0.69)	.13	3.86 (0.41)	3.79 (0.47)	.41
**Risk awareness**							
	14	3.56 (0.61)	3.64 (0.59)	.41	3.44 (0.67)	3.48 (0.66)	.52
	15	3.67 (0.53)	3.86 (0.35)	.04^a^	3.60 (0.58)	3.72 (0.63)	.20
	16	3.81 (0.40)	3.86 (0.42)	.53	3.65 (0.48)	3.72 (0.59)	.35
	17	3.58 (0.55)	3.72 (0.51)	.23	3.53 (0.55)	3.67 (0.61)	.14
	18	3.75 (0.50)	3.78 (0.42)	.76	3.44 (0.55)	3.58 (0.76)	.18
	19	2.11 (0.75)	2.47 (0.84)	.02^a^	2.05 (0.92)	2.44 (0.85)	.007^a^
**Strategies for not touching the face**							
	20	2.11 (0.98)	1.44 (0.73)	.004^a^	1.91 (0.89)	1.49 (0.69)	.002^a^
	21	3.61 (0.49)	3.72 (0.45)	.16	3.63 (0.49)	3.56 (0.73)	.82
	22	3.42 (0.65)	3.64 (0.49)	.03^a^	3.44 (0.59)	3.58 (0.66)	.23
	23	3.50 (0.61)	3.72 (0.45)	.02^a^	3.40 (0.54)	3.49 (0.63)	.25
	24	3.08 (0.81)	3.28 (0.61)	.14	2.91 (0.84)	3.23 (0.78)	.02^a^
	25	3.36 (0.59)	3.61 (0.55)	.05	3.42 (0.59)	3.56 (0.63)	.22
	26	2.58 (0.91)	2.00 (0.79)	<.001^a^	2.47 (0.91)	3.67 (0.61)	.04^a^

^a^Statistically significant differences (*P*<.05).

**Table 3:** Statistically significant differences among the control group (CG), experimental group with no additional social reinforcement (EG-nR), and experimental group with additional social reinforcement (EG-R).

Construct and item		Mann-Whitney *U* test for CG and EG-R (*P* value)	Mann-Whitney *U* test for CG and EG-nR (*P* value)	Mann-Whitney *U* test for EG-nR and EG-R (*P* value)	Mann-Whitney *U* test for CG and EG (*P* value)	Kruskal-Wallis test for CG, EG-nR, and EG-R (*P* value)
**Hygiene habits**						
	6	.69	.66	.38	>.99	.67
	7	.01^a^	.047^a^	.71	.01^a^	.04^a^
	8	.80	.43	.16	.80	.38
	9	.08	.10	.59	.06	.15
	10	.12	.81	.11	.29	.17
	11	.81	.18	.17	.39	.26
	12	.48	.34	.76	.36	.63
	13	.57	.54	.94	.50	.79
**Risk awareness**						
	14	.48	.10	.26	.20	.24
	15	.054	.007^a^	.34	.007^a^	.02^a^
	16	.14	.009^a^	.17	.02^a^	.03^a^
	17	.17	.12	.78	.10	.24
	18	.49	.11	.34	.21	.29
	19	.88	.61	.75	.73	.89
**Strategies for not touching the face**						
	20	.003^a^	.001^a^	.61	<.001^a^	.002^a^
	21	.49	.15	.43	.25	.37
	22	.06	.05^a^	.97	.03^a^	.09
	23	.62	.04^a^	.08	.18	.09
	24	.008^a^	.003^a^	.98	.002^a^	.008^a^
	25	.04^a^	.03^a^	.77	.02^a^	.051
	26	.11	.03^a^	.40	.04^a^	.08

^a^Statistically significant differences (*P*<.05).

### #RedPingüiNO Intervention

The intervention showed that 94% (74/79; EG-nR: 34/36, 94% and EG-R: 40/43, 93%) of the participants in both EGs achieved greater awareness of the times they touched their faces (item 27), and 95% (75/79; EG-nR: 34/36, 94% and EG-R: 41/43, 95%) of the participants perceived that they had managed to reduce the number of facial self-touches (item 28). There were no significant differences between both groups (*P*=.51 and *P*=.86, respectively). The intervention improved the awareness of the reasons why the participants touched their face in each situation (item 42), with a rate of 3.53 (SD 0.60) (mean 3.47, SD 0.56, for the EG-nR and mean 3.58, SD 0.63, for EG-R) on a 4-point Likert scale (1=totally disagree and 4=totally agree). Table 4 shows the results of the strategies, materials, and evaluation of the #RedPingüiNO intervention.

**Table 4:** Significant differences between experimental groups (EGs).

Item	EG, mean (SD)	EG-nR^a^, mean (SD)	EG-R^b^, mean (SD)	Mann-Whitney *U* test score for EG-nR and EG-R (*P* value)
29	3.13 (1.15)	3.33 (1.07)	2.95 (1.19)	.09
30	2.92 (1.2)	2.78 (1.24)	3.05 (1.15)	.36
31	3.05 (1.1)	3.11 (1.21)	3.00 (1.00)	.26
32	2.53 (1.34)	2.33 (1.33)	2.70 (1.34)	.25
33	2.77 (1.25)	2.44 (1.34)	3.05 (1.11)	.07
34	3.05 (1.02)	2.83 (1.13)	3.23 (0.90)	.13
35	3.04 (0.85)	3.08 (0.87)	3.00 (0.85)	.60
36	2.94 (1.23)	2.72 (1.30)	3.12 (1.16)	.18
37	3.57 (0.61)	3.44 (0.61)	3.67 (0.61)	.045^c^
38	3.48 (0.64)	3.39 (0.60)	3.56 (0.67)	.13
39	3.49 (0.62)	3.39 (0.60)	3.58 (0.63)	.10
40	3.16 (0.72)	2.89 (0.71)	3.40 (0.66)	.001^c^
41	3.63 (0.58)	3.53 (0.56)	3.72 (0.59)	.054
42	3.53 (0.60)	3.47 (0.56)	3.58 (0.63)	.27

^a^EG-nR: EG with no additional social reinforcement.

^b^EG-R: EG with additional social reinforcement.

^c^Statistically significant differences (*P*<.05).

### Strategies and Materials

On a 4-point Likert scale, where 1 was “Little” and 4 was “A lot,” the intervention groups showed high rates in the perception of the different materials, highlighting recording (31) and video (item 36) over infographics (item 32). The strategies in the game of saying “No!” (item 29) and saying “Bravo!” (item 30) had high and similar results in both the groups. The follow-up strategy (item 33) was assessed by the participants of the EG-R at a higher rate than those of the EG-nR, without it being significant (*P*=.07). The Telegram strategies with high rates in both groups also did not establish significant differences between the 2 EGs (items 34 and 35).

### Evaluation of the Intervention

The intervention was assessed by the participants of the 2 EGs as engaging (item 37), helpful (item 38), and motivating (item 39), with high rates; however, the EG-R showed a higher rate than the EG-nR in the perception of the appeal of the intervention (*P*=.045). In both groups, the duration was perceived as sufficient, with high means (item 40) and a higher rate in the EG-R than in the EG-nR (*P*=.001). In addition, the serious game proposed as an intervention strategy was perceived as suitable for use within the school context (item 41), with high rates in both groups. Therefore, although the results are satisfactory in both groups, social reinforcement seems to have an impact on the perception of the intervention with higher means in the EG-R in all cases, regardless of whether they establish significant differences. This highlights the perception of the intervention as an engaging way to train awareness to not touch the face long enough to achieve changes in this behavior.

### Development of the Daily Intervention Log

In the analysis of the development of the daily log, a significant positive difference is observed in relation to the times that the participants of both EGs consciously stopped their hand before touching their face with a “Bravo!” (EG, *P*<.001; EG-nR, *P*=.01; and EG-R, *P*<.001), as well as a significant negative difference in relation to the frequency in which the facial T-zone was touched, and they realized it with a “No!” (EG, *P*<.001; EG-nR, *P*=.049; and EG-R, *P*<.001), possibly due to the significant reduction in facial self-touches. These data must be understood as the perception of a decrease in the frequency of facial self-touches. The reasons for facial self-touches were not modified among participants throughout the intervention. The participants of the EG-nR and EG-R established the reasons for touching their face as itching, lacrimation, and tingling (EG-nR: 17/36, 47%, preintervention and 16/36, 44%, postintervention; EG-R: 26/43, 61%, preintervention and 21/43, 49%, postintervention); restlessness, discomfort, and boredom (EG-nR: 13/36, 36%, preintervention and 12/36, 33%, postintervention; EG-R: 10/43, 23%, preintervention and 9/43, 21%, postintervention); and learned gestures (EG-nR: 6/36, 17%, preintervention and 8/36, 22%, postintervention; EG-R: 7/43, 16%, preintervention and 13/43, 30%, postintervention). The motivation perceived by the participants throughout the intervention showed a significant improvement, on a 5-point Likert scale, in the EG-nR (3.47, SD 0.87 at preintervention and 4.06, SD 1.09 at postintervention) and in the full EG (3.59, SD 1.25 at preintervention and 3.93, SD 1.16 at postintervention). This difference is not established in the EG-R (3.65, SD 1.40 at preintervention and 3.86, SD 1.21 at postintervention), owing to the perceived high motivation at the start of the intervention.

## Discussion

### Principal Findings

The main objective of the #RedPingüiNO intervention was to reduce facial self-touches as a preventive measure against SARS-CoV-2 and other pathogens in various health contexts. The newly constructed tools developed for analyzing the intervention established adequate validity and reliability. After the 2-week intervention, the participants perceived improvement in their hygiene habits strategies, such as handwashing; disinfection of objects; the use of a handkerchief; and the action of avoiding touching the eyes, nose, and mouth. After the intervention, the perception of the risk that facial self-touches entail increased within the EGs, but at the same time, touching the face was noticed as an easier behavior to avoid. The participants in the 2 EGs perceived that they had reduced the number of facial self-touches following the intervention. Participants’ perception of the specific number of times they touched their faces also increased. They became more aware of the reasons why they touched their faces on a daily basis, the frequency of this behavior, and the importance of this awareness for self-regulation and avoiding self-touches. #RedPingüiNO was considered an appropriate and fun experience. More than half of the people who participated rated the game with qualifiers like “satisfactory,” “rewarding,” “positive,” “fun,” “useful,” or “surprising”; moreover, during the intervention, motivation increased as they perceived the achievement.

### Comparison With Prior Work

This study seems to reinforce that changes in health behaviors are possible with appropriate tools. This need is based on the idea that the most effective steps to reduce infection and death rates during the pandemic have been based on changes in individual behavior [2]. After the intervention, touching the face was noticed as an easier behavior to avoid, which coincides with the approach of Heinicke et al [30]. These authors indicate that reducing or stopping facial touching is a habitual and prevalent behavior that is unlikely to decrease unless we train with specific strategies for self-regulation, which implies awareness and the establishment of effective responses. Facial self-touches seem to increase in frequency and duration in emotionally or cognitively challenging social situations. This may be due to the attention states, working memory processes, and emotion-regulating functions [12] associated with such situations, which are developed and maintained based on the body language used in interactions with oneself and other people. Itching, lacrimation, and tingling were the most noted triggers of this behavior among the participants who subsequently experienced restlessness, discomfort, and boredom; the participants were somewhat less alert to the learned gestures.

The increased perception of a reduction in the number of facial self-touches seems to respond to the fact that during serious play, there was an improvement in awareness of this behavior that involved limited cognitive effort, control, and intention [31]. This can be seen in the progressive increase of “No!” in the log and how the positive reinforcement also increased with the passage of time when stopping self-touches with the “Bravo!” strategy. These game dynamics respond to the need for control over self-touches, which must be trained through habits to block, divert, or perceive the act through attention and anticipation, as well as from the reflexive capacity of movement [2]. Although we perceive the touch when it has already occurred, with repetition, there is a possibility of anticipating and paying the necessary attention to stop the action before it happens.

Although the effectiveness of strategies using mindfulness [32] or using technological means, such as software [33], camera and sensor networks [34], and smartwatches [35], to reduce facial self-touches is being investigated, specific strategies should be provided to reduce self-touches, ranging from awareness to easily understanding triggers and mechanisms to regulate one’s own behavior, if possible, from everyday contexts [8,9,15,36]. This was the case in this intervention. One participant said as follows:

The most important thing in this experience is to consider each situation that causes me to touch my face or the situations that do not. I have questioned every detail (makeup, fringe, daily face washing, piercings, glasses, hot-cold, allergies, etc.)

participant 14

Thus, awareness is understood as a mental activity of the individuals that enables them to feel, have a reflexive knowledge of their actions, and know about themselves in particular, including actions less regulated by consciousness.

This serious game was considered useful, satisfactory, and fun—an intrinsic aspect of the act of playing [37]. This shows the positive balance of game design by providing a state of well-being, pleasure, and enjoyment, as described in flow theory [38], reinforcing the notion that serious games can be a playful and effective methodological framework for learning [39,40]. In a game aimed at learning, the media and materials are fundamental and enable its development [41], and if they are technological, they can be relevant allies for interaction [42], as the highly valued video tutorials, infographics, daily logs, and the Telegram group proved to be in this study. In summary, a game like #RedPingüiNO is based on its design and implementation, and therein lies its potential, taking care of the aspects of the mechanics, dynamics, and aesthetics framework. This mechanics, dynamics, and aesthetics framework is a formal approach to understanding games, which attempts to bring together game design and development, criticism, and technical research [43].

Starting from these elements, the intrinsic motivation of the participants was sought from Marczewski theory [44], valuing autonomy in game development, the mastery that enabled the participants to be aware of the progress they were achieving, the purpose for which they were playing, and their relationships. Regarding the latter, the participants from the groups both with and without reinforcement managed to be more aware of the times they touched their faces and perceived a reduction in self-touches. However, the participants in the EG-R considered the game more appealing, believed more firmly that the allotted time was sufficient, and presented higher frequencies of self-touch awareness and restraint during the recording. In summary, the game without this accompaniment was helpful, although the collective support and reinforcements promoted among those who played it may have a relevant supporting role. Regarding playing with other people, a significant number of participants indicated that they had shared it with their families and friends, seeking out that social reinforcement themselves but within their own environment:

[W]hat has struck me the most in this intervention has been the fact that I shared it with my family. They have helped me to be much more aware of the number of times I touch my face.

participant 8

I have learned a lot, in fact, I have encouraged my family and friends to do it with me and together we have greatly reduced how much we touch our faces. When we sat down to eat, we talked about it and we kept a family ranking too.

participant 17

### Limitations and Future Directions

#RedPingüiNO resulted in a significant and helpful serious game that increased the perception of face self-touch avoidance. However, this study had some limitations. First, the sample used was a small number of university students, and it would be expedient to conduct future research with larger and more diverse samples. Second, owing to the specific characteristics of the university groups of students, there was a certain difference between the number of male and female participants in the different groups, and the CG had a smaller number of participants, which could bias the results. However, no significant gender differences were found in the study. Third, data on whether participants touched their face were not obtained from recordings. As not touching the face is a self-controlled behavior, these data were collected via a questionnaire and the daily logs of participants, and the results could be overestimated or underestimated. Therefore, as this behavior was not directly observed, these results must be understood as participants' perceptions of the decrease in the frequency of facial self-touches.

However, these limitations may present an opportunity because self-control itself has been one more strategy for reducing facial self-touches, with people taking a more active role in being aware of it and playing the game in their daily environments, not under laboratory conditions or in simulations. The perception of automated behaviors serves as a mechanism for self-control, which is facilitated by the awareness generated through conscious observation. Moreover, the perceived efficacy of the game invites to demonstrate its potential among other populations, such as health care workers and susceptible people, or within other environments.

### Conclusions

This study provides evidence on the possible impact of the serious game #RedPingüiNO in reducing the behavior and perception of touching the face and its mucous membranes and may have an effect on the prevention of infectious diseases in different health contexts, such as those caused by SARS-CoV-2 and other pathogens. Being able to exert some control over this transmission path from within a game has allowed for better conscious control beyond verbally warning people not to touch their faces. The implemented tools were considered valid and reliable. Participants perceived a reduction in facial self-touches after the intervention and became more aware of their risk behaviors. The play strategy of #RedPingüiNO is simple and motivating in its format, precisely because it deals with a complex behavior that has a high degree of unconscious movement and automatic action, which is regulated by various contextual internal and external communication situations for the individual. Challenge-oriented mechanics, levels, and humor-based onomatopoeia reinforcement, as well as accessible and free gameplay components, make this serious game adaptable to other contexts such as health institutions, hospitals, community centers, schools, or similar entities. We also noted that the game could have a greater effect if it is shared, and the experience is reinforced by the interactions between the participants. #RedPingüiNO can help control risk behaviors against infectious diseases or in exceptional health situations. In summary, this study can be presented as a precedent for the self-control of behaviors related to health at different levels.

## Appendix

Multimedia Appendix 1 Survey instrument.

## Abbreviations

CG: control group
EG: experimental group
EG-nR: experimental group with no additional social reinforcement
EG-R: experimental group with additional social reinforcement
sFST: spontaneous facial self-touches
