# Supplementary material for: Impact of a Serious Game (#RedPingüiNO) to Reduce Facial Self-Touches and Prevent Exposure to Pathogens Transmitted via Hands: Quasi-Experimental Intervention
Source: JMIR Serious Games. 2023 Jun 30;11:e45600. doi: 10.2196/45600 (PMC10365603; doi:10.2196/45600)
Supplement: Multimedia Appendix 1 [file games_v11i1e45600_app1.pdf]

Ad-hoc questionnaire about facial self-touches and the game RedPingüiNO

Ítems	TP
Me lavo diariamente las manos con una frecuencia aproximada de…	Frecuencia
Evito tocarme ojos, nariz y boca	Escala Likert 1-4
Uso pañuelo desechable cuando toso o tengo mucosidad	Escala Likert 1-4
Desinfecto las cosas que no son mías antes de tocarlas	Escala Likert 1-4
Mantengo la distancia social de 1,5 metros con las personas con las que no convivo, siempre que la situación me lo permite	Escala Likert 1-4
Uso la mascarilla fuera de casa y solo la retiro para comer, beber, fumar o hacer deporte	Escala Likert 1-4
Cuando llevo mascarilla me toco la cara con mayor frecuencia	Sí/No
Ya sabía, antes de rellenar este cuestionario, que la llamada 'zona T' de la cara es la que forman los ojos, la nariz y la boca	Sí/No
Los objetos y las superficies que toco pueden contener algún virus potencialmente nocivo para las personas	Escala Likert 1-4
Tocarme la cara es una conducta de riesgo para el contagio del virus de la COVID-19	Escala Likert 1-4
Lavarme las manos disminuye el riesgo de contagio de la COVID-19	Escala Likert 1-4
La consciencia de los auto-toques en la zona T (ojos, nariz y boca) puede reducir el número de infecciones de la COVID-19	Escala Likert 1-4
Tocarme la cara aumenta el riesgo de contagio de la COVID-19	Escala Likert 1-4
Tocarme la cara es fácil de evitar	Escala Likert 1-4
Me toco la cara con una frecuencia aproximada de…	Frecuencia
Sería deseable conocer estrategias para prevenir el contagio a través de los auto-toques en la cara	Escala Likert 1-4
El hábito de tocarme la cara se puede reducir si lo entreno	Escala Likert 1-4
Si soy consciente de cuándo me toco la cara, podría reducir el número de veces que lo hago	Escala Likert 1-4
Cuando me pica/molesta alguna zona de la cara, si cuento hasta 10 antes de tocarla, posiblemente me daría tiempo a buscar un pañuelo para aliviarme con él	Escala Likert 1-4
Si soy capaz de no tocarme la cara tras entrenarlo, sentiría satisfacción personal	Escala Likert 1-4
Me toco la cara…	Escala Likert 1-4
He logrado ser más consciente de las veces que me toco la cara	Sí/No
He logrado reducir el número de auto-toques en la cara	Sí/No
Decir "¡NO!" al tocarme la cara	Escala Likert 1-4
Decir "¡OLÉ!" al frenar y no tocarme la cara	Escala Likert 1-4
Llevar el registro diario	Escala Likert 1-4
Las infografías explicativas	Escala Likert 1-4
Llevar a cabo este programa acompañado/a de mis compañeros/as	Escala Likert 1-4
Los mensajes y refuerzo de la moderadora del grupo de Telegram	Escala Likert 1-4
Los recordatorios diarios en Telegram	Escala Likert 1-4
Los vídeos explicativos	Escala Likert 1-4
Es una forma atractiva de entrenar la consciencia de no tocarme la cara	Escala Likert 1-4
Es una forma efectiva de entrenar la consciencia de no tocarme la cara	Escala Likert 1-4
Es una intervención que me ha motivado para reducir los auto-toques en la cara	Escala Likert 1-4
Es una intervención con una duración suficiente para lograr cambios visibles en la conducta de tocarme la cara	Escala Likert 1-4
Es una estrategia adecuada para utilizar en el contexto escolar	Escala Likert 1-4
Es una intervención que me ha hecho más consciente de las razones (picor/malestar, inquietud, aburrimiento y/o gesto aprendido) por las que me toco la cara en cada situación	Escala Likert 1-4
¡Tu experiencia es muy importante! Déjanos tu comentario más personal sobre la intervención Red PingüiNO aquí:	Abierta

Abbreviations: TP= Question Type
